# Laser Management and Safety in Dermatology

**DOI:** 10.7759/cureus.25991

**Published:** 2022-06-16

**Authors:** Yasmina El Arabi, Fouzia Hali, Soumiya Chiheb

**Affiliations:** 1 Dermatology, Ibn Rochd University Hospital, Casablanca, MAR

**Keywords:** laser application, laser treatment, laser safety, laser room, lasers in dermatology

## Abstract

Lasers have acquired a wide application in dermatology due to the demand for more precise and less invasive treatments. Their indications are multiple, with proven effectiveness. Nevertheless, their use has some risks. The objective of our review is to summarize the technical and practical characteristics related to the use of lasers to ensure their management, effectiveness, and safety.

## Introduction and background

Laser is an acronym for light amplification by stimulated emission of radiation. It is a source of electromagnetic radiation capable of cutting, coagulating, destroying, or re-presenting tissues [1]. Lasers have acquired a wide and common application in dermatology due to the demand for more precise and less invasive treatments. Indeed, their indications are multiple, with proven effectiveness. Nevertheless, their use has some risks: ocular damage, burns, infection, and fire [2]. Standard norms for laser management and safety have been established by several worldwide organizations: The American National Standards Institute (ANSI), American Society for Lasers in Medicine and Surgery (ASLMS), Laser Institute of America (LIA), National Fire Protection Association (NFPA), National Institutes of Health (NIH), National Institute for Occupational Safety and Health (NIOSH), Occupational Safety and Health Administration (OSHA), and the Canadian Centre for Occupational Health and Safety (CCOHS) [3]. We summarize in this review the technical and practical features related to laser management and safety in dermatology.

## Review

Production of laser beams and their effect on the skin

Laser light is a coherent, unidirectional, intense, monochromatic wave beam. It uses wavelengths in visible light (370-750 nanometers), infrared (750-100000 nanometers), and ultraviolet (10-370 nanometers) [1]. Each wavelength has a specific receptor in the skin. In an optical cavity, each atom is stimulated to emit a pair of identical photons (stimulated emission). The amplification is done by external energy. After several round trips between the two mirrors of the optical cavity, the number of coherent photons increases (multiplication). When the signal is sufficiently intense, we have the emergence of the laser beam. Its emission can be in continuous or pulse mode [4].

The chromophores of the laser beam in the skin are water (1-10 micrometers), hemoglobin (350-460 nanometers), melanin (250-700 nanometers), and artificial pigments. Each wavelength penetrates to a specific depth. This diffusion also depends on the spot diameter, the fluence, and the pulse duration (the higher they are, the deeper the wave goes) [1,5-6].

The action can be photothermal, photomechanical, photoablation, or photochemical. The photothermal action is due to the conversion of light into heat. Several tissue damages are possible: volatilization (> 212F), evaporation (= 212F), coagulation (between 143.6 and 194F), or cell lysis (between 104 and 122F). It can produce selective photothermolysis (pulse time < thermal relaxation time) or selective photocoagulation (pulse time = thermal relaxation time). The thermal relaxation time (TRT) is defined as the time required to transfer half the received energy. The photomechanical effect is obtained when the energy of the wave is greater than the binding energy of the molecules, leading to a link rupture and the expulsion of fragments. They are very energetic lasers with very short wavelengths (excimer lasers or Q-switched lasers). Photoablation leads to a mechanical disintegration by the vaporization of water contained in tissues (CO_2_ or Erbium lasers). The photochemical action is obtained by the administration of a photosensitizer that accumulates in the target, absorbs the light, and leads to cell death or biostimulation (5 ALA, aminolevulinic acid, etc.). It is the principle of dynamic phototherapy [7].

Indications in dermatology

**Figure 1:**
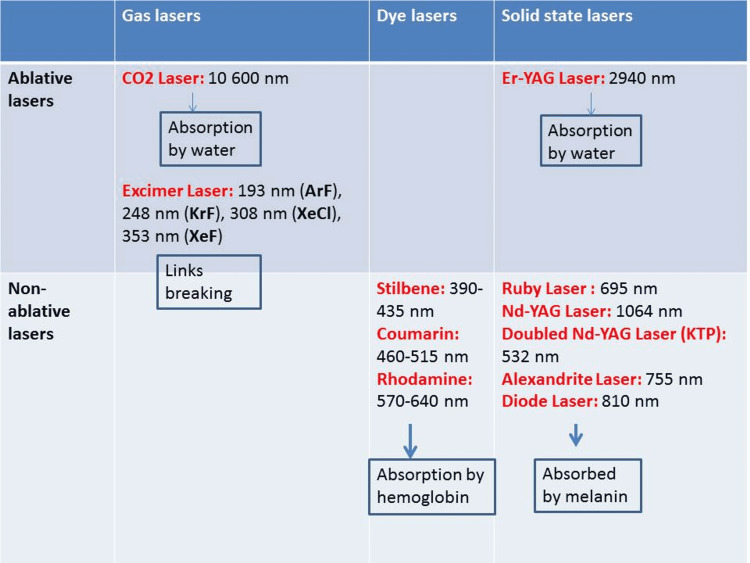
Different lasers used in dermatology

The main lasers used in dermatology are shown in Figure 1. Vascular lasers target hemoglobin. For superficial vessels, potassium titanyl phosphate (KTP), pulsed dye laser, solid yellow laser, intense polychromatic light (IPL) 540-1000 nm, or optimized pulsed light (OPL) 760 nm can be used. For deep vessels, IPL, OPL 870 nm, and neodymium-doped yttrium aluminum garnet (Nd: YAG) 1064 nm can be used. The spot diameter can vary from 1.5 to 10 millimeters. For photocoagulation, the fluence should be less than 600 J/cm² and the pulse time longer than 10 milliseconds. The endpoint is without purpura. For photothermolysis, the fluence should be higher than 600 J/cm² and the pulse time lower than 10 milliseconds. The endpoint will be with purpura. The main indications in dermatology are erythro-couperosis, erythrose colli, telangiectasias, varicosities, angiokeratoma, angioma, venous lake, residual lesion of hemangioma, inflammatory scars, and the vascular component of melasma [8-10].

Pigment lasers target melanin or artificial pigments. The devices used are Q-switched lasers with a very short pulse time. The main indications in dermatology are tattoos, Ota nevi, Becker nevi, lentigos, actinic keratosis, and café au lait spots [8-9,11].

Hair removal lasers target the hair melanin in its anagen phase, resulting in selective photothermolysis. The devices used are Alexandrite LP 755 nm (light phototypes), Nd: YAG LP 1064 nm (dark phototypes, tanned skin, deep hair), Diode 800 nm (medium phototypes), and IPL 590-1200 nm (light phototypes). The main indications in dermatology are pseudofolliculitis, hirsutism, Becker's hamartoma, suppurative hidradenitis, keloid acne, and aesthetics [6].

Ablative lasers emit beams absorbed by water. In continuous mode under local anesthesia (CO_2_ laser), it causes tissue volatilization. In fractionated mode (pulsed CO_2_ laser and Er: YAG), it causes a moderate loss in the epidermis and dermis with an interval of healthy skin resulting in skin tension and collagen regeneration [12]. The principle of non-ablative fractional lasers is to perform thermal micro-coagulation columns in the dermis. The collagen is denatured and favors the regeneration process, resulting in smoothing and rejuvenation of the skin. The devices used in fractional mode are Nd: YAG 1440 nm + IPL, Erbium 1540 nm XD XF, Erbium glass 1540 nm, Erbium: YAG 1550 nm/1927 nm, and IPL 850 nm - 1350 nm [13].

Risks associated with the use of lasers

The classification of lasers according to the risks engaged is represented in Table 1. The maximum permissible exposure is the maximum level of laser radiation to which people can be exposed without suffering from immediate or long-term damages. The main risks are ocular (corneal burns, keratoconjunctivitis, and cataracts) or cutaneous (hyperpigmentation, achromia, atrophic or hypertrophic scars, scabs, epidermal bubbles, herpes, psoriasis, or eczema). The main precautions to take are wearing glasses and gloves, a laser room that meets safety standards, avoiding sun exposition, isotretinoin, and betacarotene, avoiding in pregnant women, cleaning the skin thoroughly before the session, and applying a healing cream and sun protection after it [14].

**Table 1:** Classification of lasers according to the NF EN 60825-1/A2 standard of January 2006 (standard applicable for devices put on the market since this date)

Class 1	Lasers considered safe under all reasonably foreseeable conditions of use
Class 1M	Lasers whose direct vision in the beam, especially with optical instruments, can be dangerous.
Class 2	Lasers that emit visible radiation in the wavelength range from 400 nm to 700 nm. The protection of the eye is normally ensured by the palpebral reflex.
Class 2M	Lasers that emit visible radiation in the wavelength range from 400 nm to 700 nm and whose direct vision in the beam, especially with optical instruments, can be dangerous.
Class 3R	Lasers where the direct view of the beam is potentially dangerous but the level of risk remains lower than that of class 3B lasers.
Class 3 B	Lasers whose direct vision of the laser beam is always dangerous. The vision of diffuse reflections is normally safe.
Class 4	Lasers that are dangerous when viewed directly but are also capable of producing dangerous diffuse reflections. They can cause skin lesions and constitute a fire hazard. Their use requires extreme precautions.

Personal protection standards

The retina and the choroid strongly absorb visible light radiation, specifically ultraviolet and infrared rays. They can cause keratoconjunctivitis or cataracts, hence the interest in wearing appropriate protective eyewear.

The laser hazards to the skin are due to its thermal effect. Ultraviolet and infrared rays are the most aggressive because they are strongly absorbed at the skin surface. Exposure to these radiations with short pulses of high power can cause superficial carbonization of the epidermis, phlyctens, or erythema. Therefore, it is preferable to wear non-flammable gloves.

Ablative lasers vaporize the tissues with the emission of fumes that contain bacteria or viruses (Escherichia coli, Staphylococcus aureus, Papilloma human virus, human immunodeficiency virus, etc.), cell debris, gases (benzene, formaldehyde, hydrogen cyanide, etc.), or toxic aerosols. They must be eliminated by suction devices: air filters or fume extractors. The practitioner must also wear a respiratory protection mask (FFP2, KN95, etc.) [15-16].

Room standards

a) Surfaces

The laser room shall have no reflective surfaces to avoid accidental scattering of a laser beam. Walls should be matte painted or covered with dark blue ceramic tile, the floor covered with gray linoleum or Gerflex, and windows covered with galvanized steel.

b) Doors and Windows

These shouldn’t be located in the path of a laser beam to avoid accidental projection into a third person.

c) Lighting

Good lighting is necessary to reduce pupil dilation.

d) Equipment

Examination table: The examination table must support a weight of at least 150 kilograms and be easily stabilized or mobilized. 

A gas extinguisher: This should be present in case of fire.

Voltage stabilizer: The production of a laser beam requires the use of an energy source that will generate stimulated emission and population inversion. It is often an electrical source. While the electrical/optical efficiency of laser sources is low, the laser beam with high power requires an electrical pumping source delivering several hundred watts. Because of the electrical dangers involved, it is necessary to use a water cooling system, which must be isolated from the electrical source. Thus, a voltage stabilizer is used.

An emergency cart: This includes a defibrillator, oxygen cylinder, epinephrine, atropine, lidocaine, benzodiazepine nitrates, oxygen masks, material for intravenous or intramuscular injections, sterile compresses, non-sterile gloves, protective glasses, and antiseptic solution.

A hazard sign on the entrance door: This is usually given with the laser.

An airlock: [2-3,17-19].

Cleanliness and asepsis standards

A clean room is defined as a room in which the concentration of airborne particles is controlled and which is managed to minimize the introduction, generation, and retention of particles (ISO 14644). It should have smooth walls and floors and be regularly maintained. It also should have a hydroalcoholic dispenser, a garbage collector, and an autoclave [16].

Management structure

The management of a laser room requires the appointment of two people in charge, a unit director and a referent, whose mission is the training and continuing education of the personnel, providing the risk and prevention booklet, providing the equipment, and performing the activity inventory [20].

## Conclusions

Lasers have revolutionized the treatment of dermatology patients. Nevertheless, their use isn’t without risk. Standard norms have been established to ensure their effectiveness and safety. These norms can be summarized in the following points: a laser room with non-reflective surfaces, danger signs, choosing the right indication for use, and wearing protective equipment.
